# Nucleobase-Interaction-Directed Biomimetic Supramolecular Self-Assembly

**DOI:** 10.1021/acs.accounts.2c00135

**Published:** 2022-06-07

**Authors:** Amrita Sikder, Cem Esen, Rachel K. O’Reilly

**Affiliations:** † School of Chemistry, University of Birmingham, Edgbaston, Birmingham B15 2TT, U.K.; ‡ Department of Chemistry, Faculty of Arts and Sciences, Aydın Adnan Menderes University, 09010 Aydın, Turkey

## Abstract

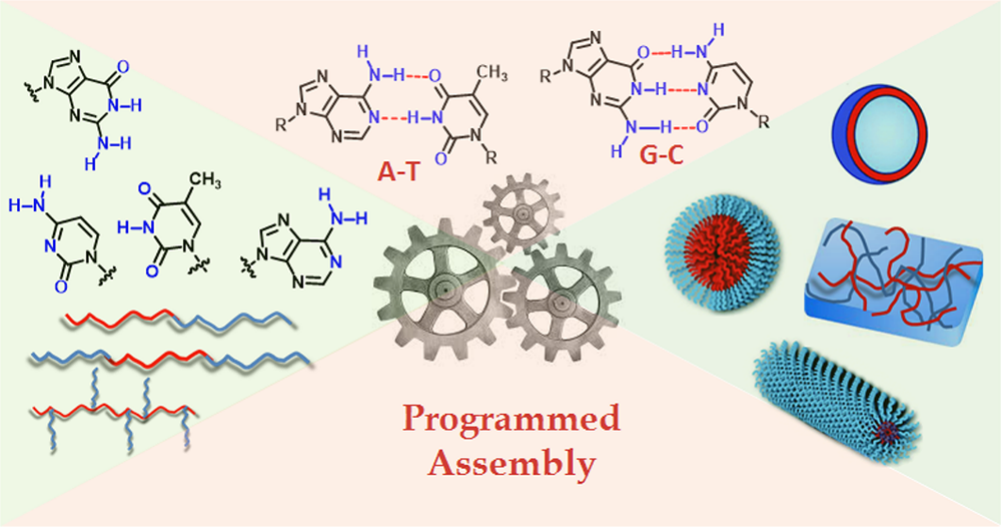

The design and fabrication of synthetic self-assembled systems that can mimic some biological features require exquisitely sophisticated components that make use of supramolecular interactions to attain enhanced structural and functional complexity. In nature, nucleobase interactions play a key role in biological functions in living organisms, including transcription and translation processes. Inspired by nature, scientists are progressively exploring nucleobase synthons to create a diverse range of functional systems with a plethora of nanostructures by virtue of molecular-recognition-directed assembly and flexible programmability of the base-pairing interactions. To that end, nucleobase-functionalized molecules and macromolecules are attracting great attention because of their versatile structures with smart and adaptive material properties such as stimuli responsiveness, interaction with external agents, and ability to repair structural defects. In this regard, a range of nucleobase-interaction-mediated hierarchical self-assembled systems have been developed to obtain biomimetic materials with unique properties. For example, a new “grafting to” strategy utilizing complementary nucleobase interactions has been demonstrated to temporarily control the functional group display on micellar surfaces. In a different approach, complementary nucleobase interactions have been explored to enable morphological transitions in functionalized diblock copolymer assembly. It has been demonstrated that complementary nucleobase interactions can drive the morphological transformation to produce highly anisotropic nanoparticles by controlling the assembly processes at multiple length scales. Furthermore, nucleobase-functionalized bottle brush polymers have been employed to generate stimuli-responsive hierarchical assembly. Finally, such interactions have been exploited to induce biomimetic segregation in polymer self-assembly, which has been employed as a template to synthesize polymers with narrow polydispersity. It is evident from these examples that the optimal design of molecular building blocks and precise positioning of the nucleobase functionality are essential for fabrication of complex supramolecular assemblies. While a considerable amount of research remains to be explored, our studies have demonstrated the potential of nucleobase-interaction-mediated supramolecular assembly to be a promising field of research enabling the development of biomimetic materials.

This Account summarizes recent examples that employ nucleobase interactions to generate functional biomaterials by judicious design of the building blocks. We begin by discussing the molecular recognition properties of different nucleobases, followed by different strategies to employ nucleobase interactions in polymeric systems in order to achieve self-assembled nanomaterials with versatile properties. Moreover, some of their prospective biological/material applications such as enhanced drug encapsulation, superior adhesion, and fast self-healing properties facilitated by complementary nucleobase interactions are emphasized. Finally, we identify issues and challenges that are faced by this class of materials and propose future directions for the exploration of functional materials with the aim of promoting the development of nucleobase-functionalized systems to design the next generation of biomaterials.

## Key References

- VarlasS.; HuaZ.; JonesJ. R.; ThomasM.; FosterJ. C.; O’ReillyR. K.Complementary Nucleobase Interactions Drive the Hierarchical Self-Assembly of Core–Shell Bottlebrush Block Copolymers toward Cylindrical Supramolecules. Macromolecules2020, 53, 9747–9757.^1^*This article reports thermally induced supramolecular assembly of bottlebrush polymers facilitated by complementary H-bonding between nucleobase pairs.*
- HuaZ.; JonesJ. R.; ThomasM.; ArnoM. C.; SouslovA.; WilksT. R.; O’ReillyR. K.Anisotropic polymer nanoparticles with controlled dimensions from the morphological transformation of isotropic seeds. Nat. Commun.2019, 10, 54063177633410.1038/s41467-019-13263-6PMC6881314.^2^*This communication reveals a method to produce highly anisotropic nanoparticles with controlled dimensions by means of a morphological transformation process driven by complementary nucleobase interactions.*
- HuaZ.; KeoghR.; LiZ.; WilksT. R.; ChenG.; O’ReillyR. K.Reversibly Manipulating the Surface Chemistry of Polymeric Nanostructures via a “Grafting To” Approach Mediated by Nucleobase Interactions. Macromolecules2017, 50, 3662–36702852938210.1021/acs.macromol.7b00286PMC5435456.^3^*This article describes a supramolecular “grafting to” method to fabricate highly functionalized mixed polymeric nanostructures exploiting multiple H-bonding interactions between thymine- and adenine-containing complementary polymers.*
- HuaZ.; Pitto-BarryA.; KangY.; KirbyN.; WilksT. R.; O’ReillyR. K.Micellar nanoparticles with tunable morphologies through interactions between nucleobase containing synthetic polymers in aqueous solution. Polym. Chem.2016, 7, 4254–426210.1039/c6py00263cPMC489407327358655.^4^*This article highlights the role of complementary nucleobase interactions to induce nanostructure reorganization to achieve different nanostructures of variable shape and size.*

## Introduction

**Scheme 1:**
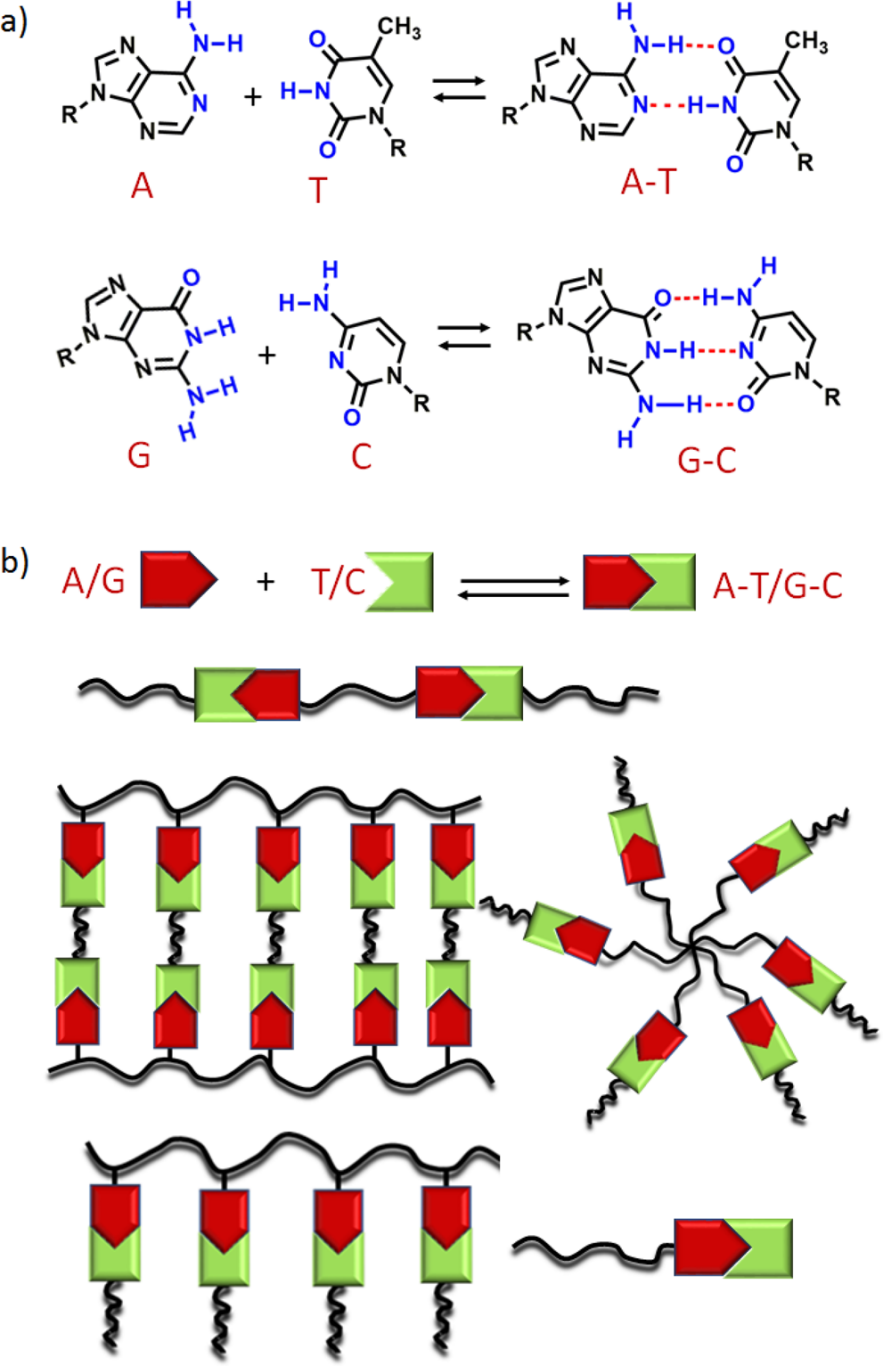
(a) Chemical Structures of the Natural Nucleobases A, T, G, and C and Their Modes of Molecular Recognition by Complementary H-Bonding; (b) Schematic Representation of Various Topologies of Base-Pair-Interaction-Mediated Supramolecular Assembly

DNA base pairing is considered to be the blueprint of life, where the nucleobase interactions serve to underlie the chemistry because of their high fidelity, molecular recognition ability, sequence specificity, and directionality. Since the elucidation of the DNA double helix structure, bound together through complementary H-bonding between adenine (A) and thymine (T) and between guanine (G) and cytosine (C),^5^ nucleobase interactions have been widely explored. The development of highly organized biomimetic materials through the programmed assembly of bioinspired molecular building blocks has been a rapidly expanding field in the areas of functional materials, biomedicine, and bioengineering processes.^6,7^ The rich chemistry between nucleobase pairs with unique features such as reactivity, responsiveness, chirality transfer, and biocompatibility as well as their inexpensive commercial availability has motivated scientists to use nucleobase functionality in molecular and macromolecular assembly.^8−12^ Exploiting nucleobases to generate functional materials can offer flexibility because of the different binding properties of different nucleobases. For example, the A:T base pair interacts via two-point H-bonding with a binding constant (*K*_a_) of ∼10^2^ (in chloroform), whereas the G:C pair involves three-point H-bonding with a 2 orders of magnitude increase in binding constant (*K*_a_ ∼ 10^4^ in chloroform) (Scheme 1a).^13^ Interestingly, this is not the exclusive mode of interaction between these nucleobases, as there are other 28 possible patterns^14^ of base pairs involving at least two H-bonds that can be formed between these nucleobases.

Although H-bonding interactions primarily control the selectivity of these complementary-base-pair interactions, other non-covalent interactions such as hydrophobic interactions, aromatic interactions, and electrostatic interactions also play a crucial role in regulating the morphology and functionality of nanostructures.^15−18^ Therefore, the possibilities are endless for creating elegant nanostructures by designing suitable building blocks with different topologies (Scheme 1b) as well as by introducing additional non-covalent interactions along with base-pair interactions. In the following sections, we highlight and discuss several recent examples that employ supramolecularly engineered nucleobase precursors to generate functional biomaterials with unique properties.

## Nucleobase-Interaction-Mediated Hierarchical Organization of Polymers

Polymer nanostructures generated by nucleobase-interaction-mediated bonding are of great prominence, as the main features of the native polymer such as physical and mechanical properties are retained, in addition to the advantage of smart changing of properties in the presence of external stimuli, making them a novel class of smart materials. Significant advances in the synthesis of nucleobase-functionalized polymers were made in the early 1990s with the preparation of methacrylate monomer analogues.^19^ Subsequently, with the development of various polymerization techniques, many efforts have been made toward the synthesis of nucleobase-containing polymers and to explore the effect of complementary nucleobase interactions on supramolecular assembly properties.^20−28^

**Figure 1:**
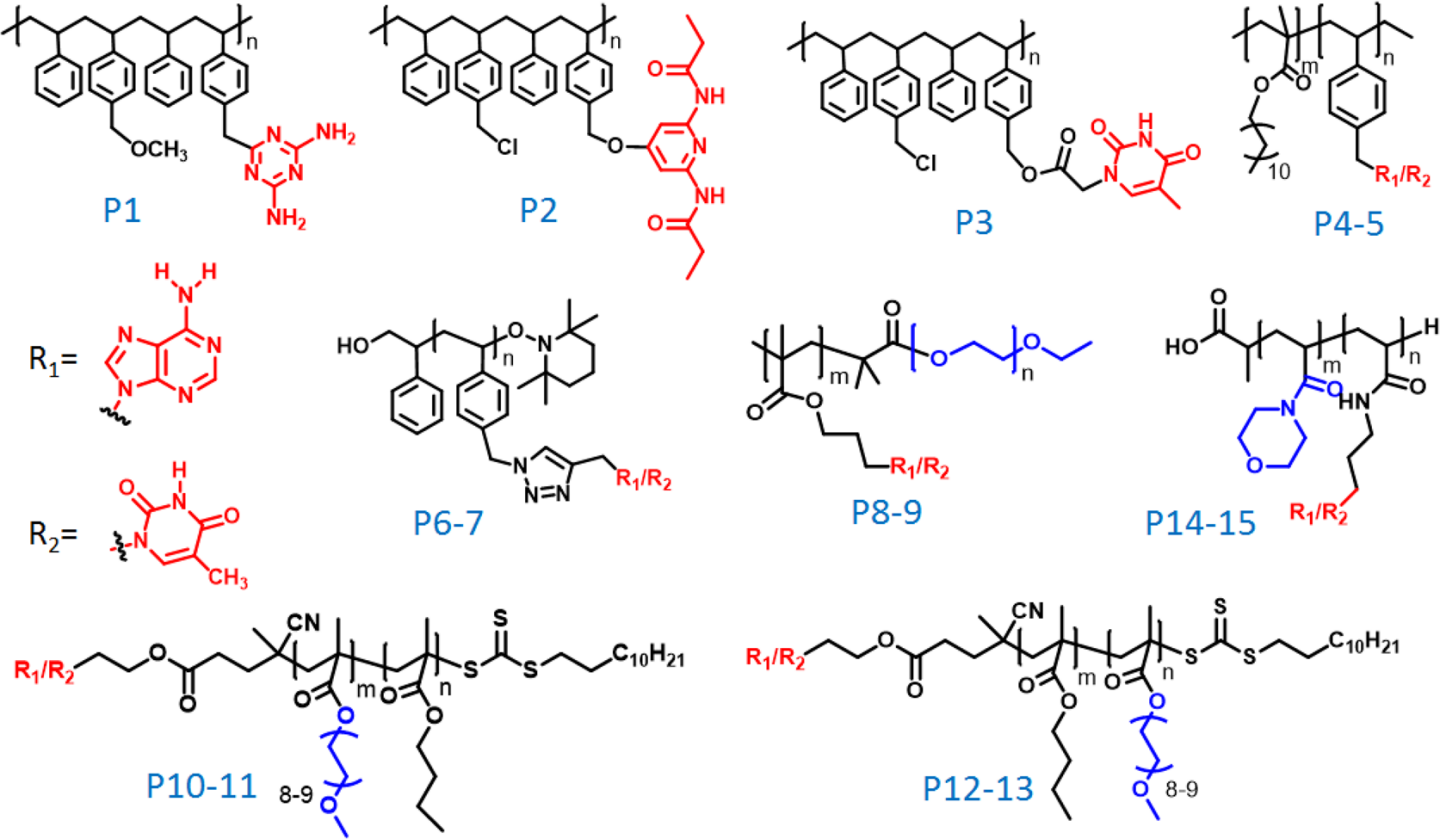
Structures of different nucleobase-functionalized polymers discussed in this Account.

Rotello and co-workers^20^ first demonstrated the involvement of complementary H-bonding in nucleobase analogues in order to form a highly ordered assembly via molecular recognition. Assembly of a diaminotriazine-functionalized polymer (P1 in Figure 1) with a complementary uracil-containing guest molecule produced a guest-encapsulated micelle via selective intermolecular H-bonding between the diaminotriazine and uracil units. Subsequently, they reported an unprecedented method of supramolecular vesicle formation by coassembly of complementary-nucleobase-functionalized random copolymers (P2 and P3 in Figure 1).^21,22^

Early work involving nucleobase-mediated polymer assembly was reported by Lutz et al.^23,24^ They synthesized random copolymers using dodecyl methacrylate (DMA) and vinylbenzyl derivatives of the nucleobases adenine and thymine (VBA/VBT). The supramolecular coassembly of VBA-*co*-DMA and VBT-*co*-DMA (polymers P4 and P5 in Figure 1) was studied in various organic solvents with different polarities to realize the effect of the solvent on A:T interactions.^23^ They also revealed “DNA-like” melting behavior of a 1:1 coassembly of VBA-*co*-DMA and VBT-*co*-DMA that was absent in the corresponding self-assemblies of the individual polymers.^24^ In a different design, Kuo and co-workers^25^ explored the effect of nucleobase interactions on polymer assembly by synthesizing adenine/thymine-functionalized poly(vinylbenzyl) polymers (PVBA/PVBT) of the same molecular weight by postpolymerization modification (polymers P6 and P7 in Figure 1). Comixing of PVBA and PVBT in DMF solution yielded spherical aggregates with high thermal stability through A:T complementary H-bonding interactions.

**Figure 2:**
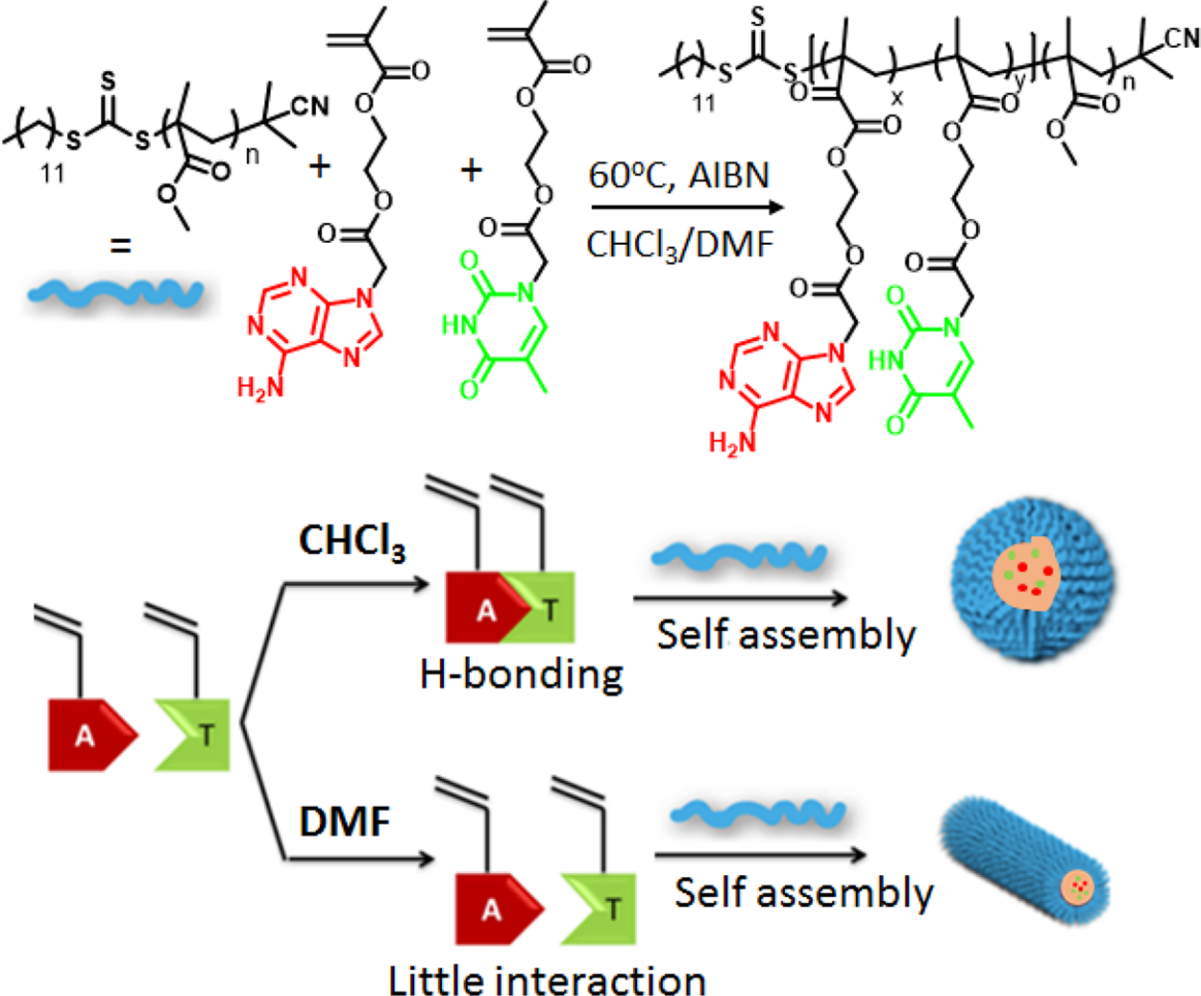
Schematic representation of the synthesis and self-assembly of nucleobase-functionalized block copolymers. Adapted from ref (26). Copyright 2013 American Chemical Society.

Our group has explored the effect of the complementary interaction strength on copolymerization of nucleobase-functionalized methacrylate monomers.^26^ Copolymerization in chloroform (CHCl_3_) led to an alternate copolymer because of the strong intermolecular H-bonding between the adenine and thymine monomers, whereas in dimethylformamide (DMF) statistical copolymers were formed because of the absence of nucleobase interactions. Interestingly, self-assembly of the block copolymers prepared in DMF and CHCl_3_ led to distinctly different morphologies depending on the polymerization conditions, further highlighting the role of nucleobase interactions in tuning hierarchical assembly (Figure 2).

Apart from linear polymer chains, nucleobase-containing star-shaped polymers have also been explored to investigate the effect of complementary nucleobase interactions. Long and co-workers^27^ prepared four-arm poly(d,l-lactide) (PDLLA) polymers containing peripheral A and T units (PDLLA-A and PDLLA-T) and PDLLA without the nucleobase units as a control system. Association of the nucleobase-functionalized star-shaped polymers in CHCl_3_ resulted in higher viscosity compared with the coassembly of the corresponding non-nucleobase-functionalized precursors, confirming the formation of a supramolecular structure. Job’s plots and Benesi–Hildebrand analysis suggested the formation of a 1:1 complex with strong intermolecular H-bonding. To investigate further the effect of molecular weight on complementary recognition, a series of PDLLA-A and PDLLA-T polymers were prepared by variation of the chain length, and it was found that the molecular recognition property of the A:T unit decreased with increasing molecular weight.

So far in this Account, the supramolecular assembly of polymers in organic solutions has been discussed. Van Hest and co-workers^29^ explored the assembly of amphiphilic nucleobase-containing polymers in aqueous media. Block copolymers (PEG-*b*-A/PEG-*b*-T) (polymers P8 and P9 in Figure 1) were synthesized using poly(ethylene glycol) (PEG) as the hydrophilic block and nucleobase (A or T)-functionalized poly(methyl methacrylate) (PMMA) as the hydrophobic block. The critical aggregation constant (CAC) of the supramolecular micelles formed by complementary interactions between PEG-*b*-A and PEG-*b*-T was found to be higher than that of micelles prepared from the individual polymers because of the enhanced hydrophilicity imparted to the PMMA core as a result of A:T H-bonding.

**Figure 3:**
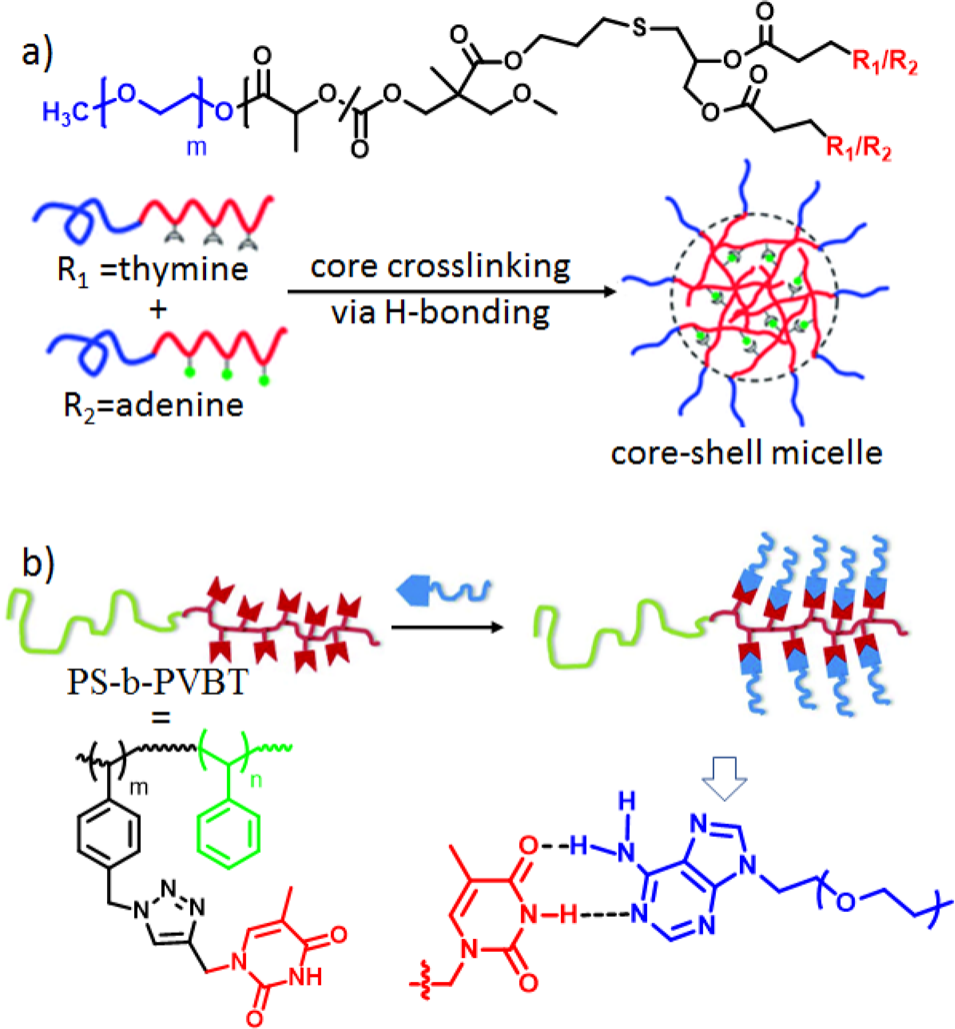
Cartoon representations of (a) core-cross-linked polymer micelle formation and (b) supramolecular graft copolymer formation. Adapted with permission from (a) ref (30) and (b) ref (35). Copyright 2012 and 2015, respectively, Royal Society of Chemistry.

Complementary H-bonding interactions have been also utilized to improve the self-assembly properties of nucleobase-functionalized polymers. For instance, Huang and co-workers^30^ synthesized nucleobase-functionalized methoxy-poly(ethylene glycol)-*b*-poly(l-lactide-*co*-2-methyl-2-allyloxycarbonylpropylene carbonate) amphiphilic block copolymers where the A and T units were present in the hydrophobic block (Figure 3a). A 1:1 mixture of the complementary polymers was found to form core-cross-linked micelles via A:T interactions exhibiting higher stability and lower CAC compared with the non-cross-linked micelles in the absence of the complementary polymer. They hypothesized that the hydrophobic microenvironment of the core promoted stronger A:T intermolecular H-bonding, leading to a significant enhancement of the micellar stability.

In a relevant work, Thang and co-workers^31^ studied the effect of the position of the nucleobase unit in a polymer chain on the complementary molecular recognition properties. They synthesized amphiphilic block copolymers composed of hydrophilic oligo(ethylene glycol) methyl ether methacrylate and hydrophobic *n*-butyl methacrylate connecting an A or T unit either at the hydrophilic end (polymers P10 and P11 in Figure 1) or at the hydrophobic end (polymers P12 and P13 in Figure 1) of the polymers. Accordingly, P10 and P11 produced micelles containing the nucleobases in the core, whereas for P12- and P13-derived micelles the nucleobases were exposed at the corona. Free bases were added to these preformed micelles in order to evaluate the complementary interactions in buffer solutions. It was observed that the base pairing was only effective for polymers P10 and P11 because of the facile access to the nucleobase units that were exposed at the outer shell of the micelles, whereas for P12 and P13 micelles the sequestration of the nucleobase functionality in the hydrophobic core did not allow molecular recognition with the externally added guest.

Another novel approach to form supramolecular nanostructures is to impart amphiphilicity in the polymer chains by harnessing the nucleobase interactions. For example, Deng and co-workers^32^ synthesized adenine-modified poly(ε-caprolactone) (PCL) and thymine-modified PEG and prepared nanoparticles in situ by comixing the polymers. Moreover, they highlighted the advantage of complementary base pairing to achieve polymer micelles with a very narrow size distribution compared with conventional amphiphilic assembly of diblock polymers. By the same principle, water-soluble luminescent nanoparticles were prepared by coassembly of thymine-functionalized π-conjugated fluorescent polymers with adenine-containing PEG.^33^ In this regard, we have reported a unique way to introduce fluorescence in a novel class of nucleobase-containing diblock copolymers containing an adenine or thymine unit as the hydrophobic block and polymorpholine as hydrophilic segment (polymers P14 and P15 in Figure 1).^34^ A 1:1 assembly of the complementary polymers produced a non-fluorescent core–shell supramolecular micelle, whereas photo-cross-linking of the thymine units followed by rigidification and immobilization of the adenine units due to extensive H-bonding at the core imparted fluorescence to the polymer nanoparticles.

Apart from supramolecular block copolymers, the assembly of nucleobase-functionalized graft polymers has also been explored. Kuo and co-workers^35^ reported the formation of multicompartment micelles of different shapes, from raspberry-like spheres to core–shell cylinders to trilayer vesicles, by grafting adenine-terminated PEG onto the thymine-functionalized block copolymer poly(styrene-*b*-4-vinylbenzyl triazolylmethyl methylthymine) (PS-*b*-PVBT) as a function of the DMF:H_2_O ratio as well as the molecular weight of the PS-*b*-PVBT block (Figure 3b).

## Nucleobase-Mediated Morphological Evolution

There has been growing interest in controlling and selectively tuning nanostructure morphology, as smart functionalities can be imparted in polymer nanoparticles by regulation of their sizes and shapes. For example, cylindrical micelles have a longer circulation lifetime as drug delivery vehicles compared with spherical micelles, and short cylinders are more prone to cellular uptake than longer cylinders.^36^ Therefore, there is always an urge to develop tunable systems via a programmable hierarchical assembly strategy. One effective way of achieving this is by tailoring the non-covalent interactions in self-assembled nanostructures.

**Figure 4:**
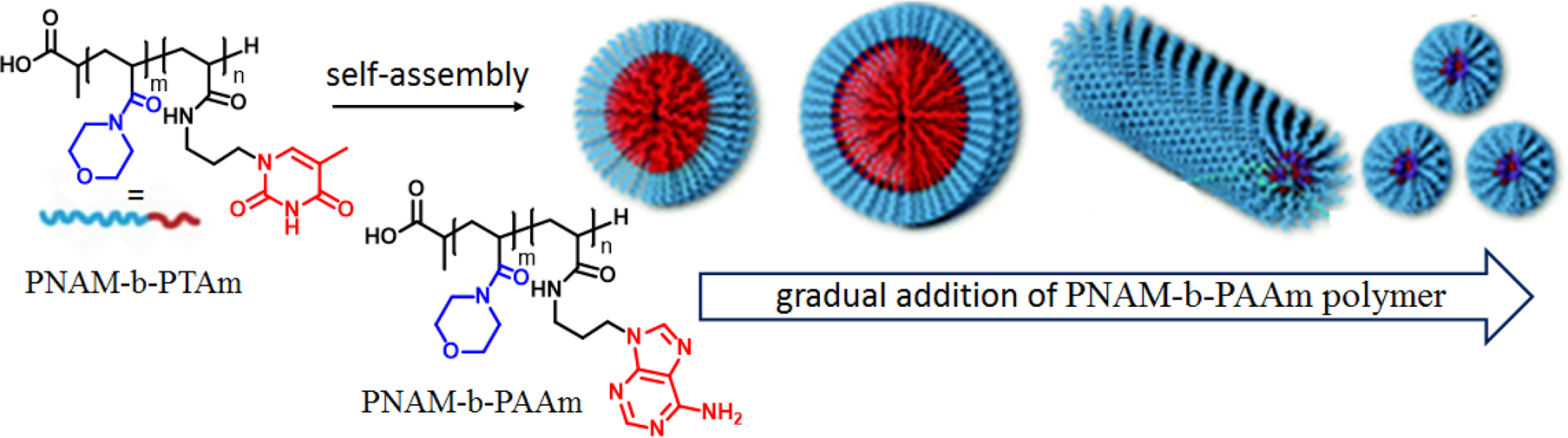
Schematic representation of morphological evaluation of thymine-functionalized diblock copolymer micelles in the presence of complementary adenine-functionalized polymer. Adapted with permission from ref (4). Copyright 2016 Royal Society of Chemistry.

With this aim, we have explored nucleobase-functionalized complementary block copolymers consisting of polyacryloylmorpholine (PNAM) blocks and either A- or T-conjugated polypropylacrylamide blocks (PNAM-*b*-PAAm and PNAM-*b*-PTAm) to tune the size and morphology of the polymer nanostructures (Figure 4).^4^ PNAM-*b*-PTAm formed a micellar assembly in which the core consisted of thymine units, and addition of PNAM-*b*-PAAm formed A:T H-bonding networks, resulting in an initial increase in the size of the micelles due to the high energy barrier of the chain exchange of the long hydrophobic block. With gradual addition of the complementary polymer both the corona-chain repulsion and core-chain stretching were reduced, which led to a morphological transformation to cylindrical nanostructures with subsequent disassembly to form smaller micelles upon further addition of PNAM-*b*-PAAm. Interestingly such morphological transformations were found to be dependent on the hydrophobic block length of the PNAM-*b*-PTAm polymer.

We have further demonstrated controlled growth of polymer nanoparticles that exploits base-pair interactions to produce anisotropic structures with a high aspect ratio.^2^ Here we have proposed a model based on a morphological transformation process (MORPH) that utilizes A:T H-bonding interactions to drive the insertion of the complementary polymer into an isotropic polymer nanoparticle followed by elongation to produce wormlike nanostructures. The dimensions of the worms were dependent on the amount of complementary polymer added. It is worth noting that the size of the worm and the amount of complementary polymer added displayed a linear relationship, which allowed us to get a desired particle of a specific length on demand.

**Figure 5:**
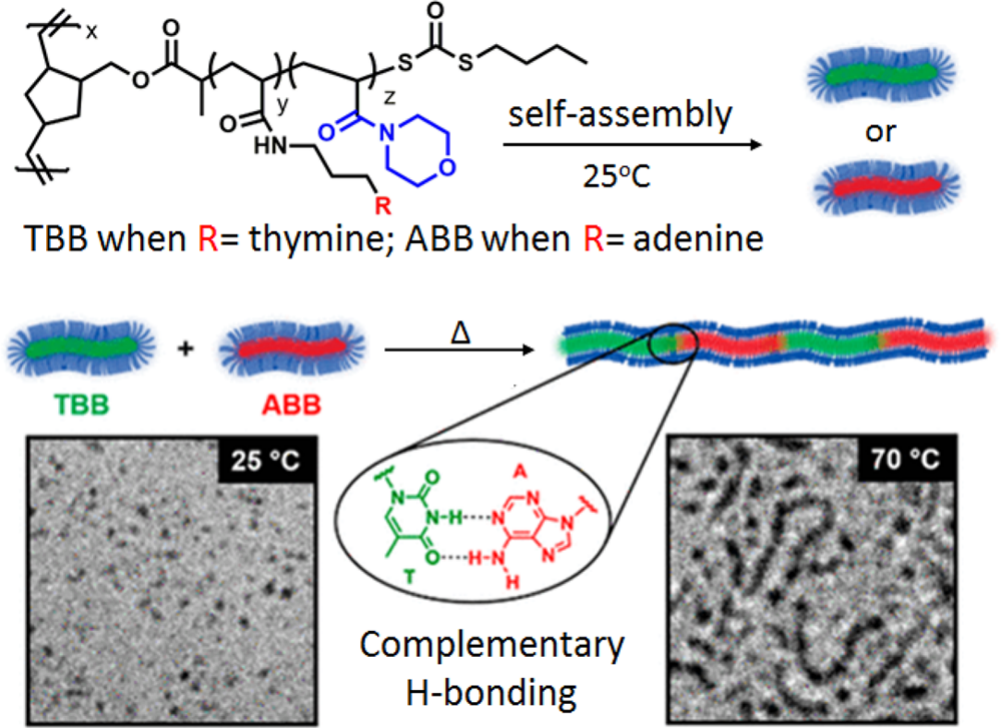
Schematic representation of end-to-end supramolecular coassembly of amphiphilic bottlebrush copolymers driven by complementary T:A H-bonding interactions. Adapted from ref (1). Copyright 2020 American Chemical Society.

Using a different design, we have also synthesized nucleobase-functionalized bottlebrush polymers to evaluate the effect of polymer topology on complementary H-bonding interactions.^1^ Thymine- and adenine-containing bottlebrush polymers (TBB and ABB) were found to form anisotropic nanoparticles individually in aqueous solution with corona-forming poly(4-acryloylmorpholine) and core-forming poly(thymineacrylamide) (TBB polymer) or poly(adenineacrylamide) (ABB polymer) blocks (Figure 5). Mixing the anisotropic structures of ABB and TBB did not alter the morphology at room temperature, as the adenine and thymine units were sequestered individually in the hydrophobic cores of the core–shell structures. However, above the lower critical solution temperature (LCST) of the poly(4-acryloylmorpholine) chain, the hydrophilic shell collapsed, facilitating close interactions between the complementary nucleobases at the edges of the anisotropic particles, leading to the formation of hierarchically assembled cylindrical micelles.

Along with A:T interactions, G:C-interaction-induced morphological evolution has been explored by Thang and colleagues.^37^ A series of amphiphilic block copolymers of similar molecular weight were prepared using different nucleobase (A/T/G/C)-functionalized styrene or methacrylate and *N*-isopropylacrylamide (NIPAM) monomers having flexible (methacrylate) or rigid (polystyrene) backbones. Notably, spherical nanoparticles were obtained for individual polymer assembly, whereas very different nanostructures were observed for 1:1 coassembly of complementary polymers, such as spindlelike structures or short rodlike morphology depending on the base pair (A:T or G:C) and the rigidity of the polymer backbone.

## Nucleobase-Mediated Non-covalent Surface Functionalization

**Figure 6:**
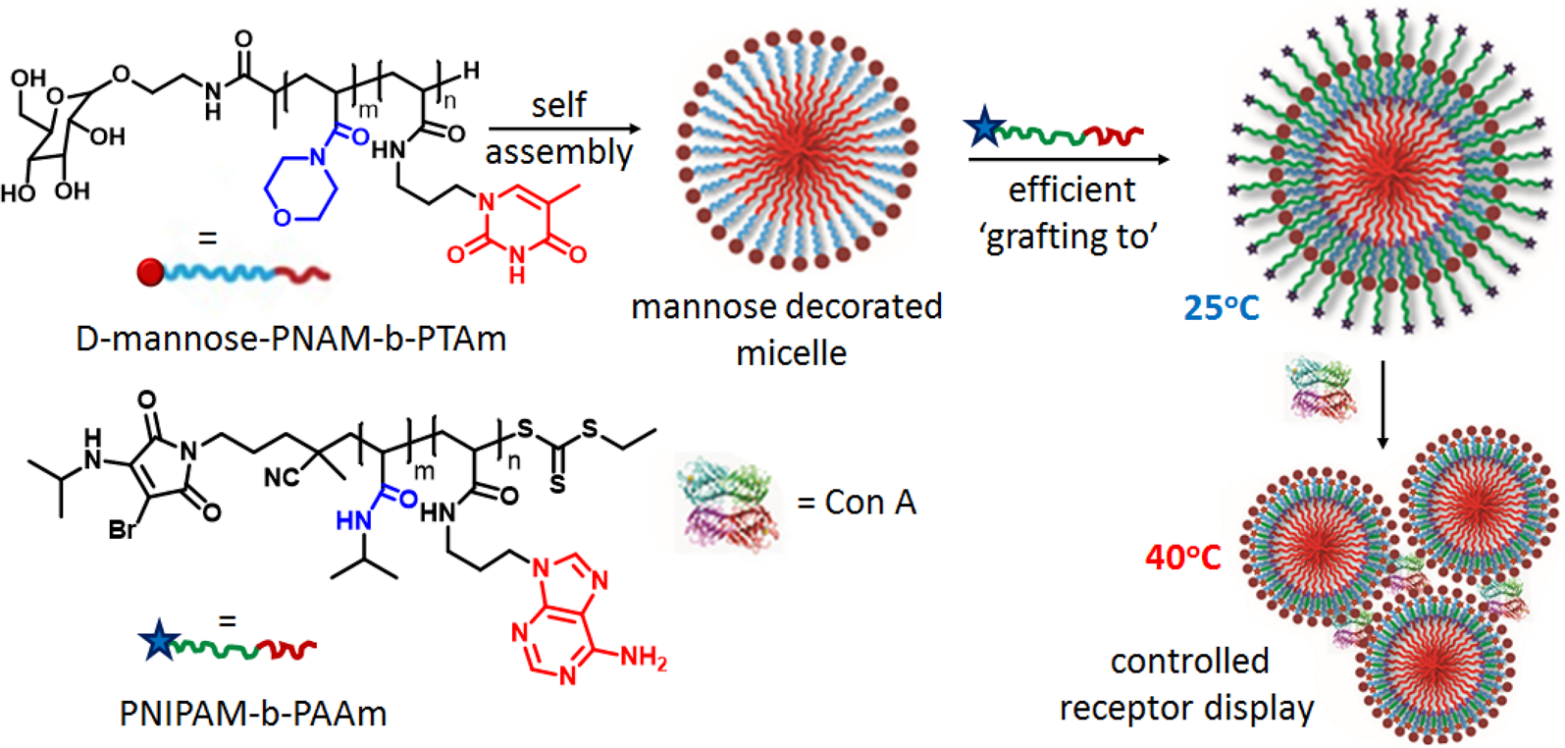
Schematic representation of the supramolecular “grafting to” approach to prepare surface-functionalized polymer micelles. Adapted from ref (3). Copyright 2017 American Chemical Society.

Along with inducing morphological changes, nucleobase interactions have been utilized for surface functionalization to impart desired properties to polymer nanoparticles. During the past decade, we have demonstrated a versatile “grafting to” strategy to prepare functionalized mixed-corona micelles by exploiting A:T H-bonding between complementary diblock copolymers (Figure 6).^3^ The thymine-containing diblock copolymer PNAM-*b*-PTAm was used to prepare core–shell micelles with a T-containing core and poly(4-acryloylmorpholine) corona. We successfully obtained stimuli-responsive micelles with different sizes by adding a series of complementary diblock copolymers (PNIPAM-*b*-PAAm) containing thermoresponsive (PNIPAM) blocks with various chain lengths. Next, we targeted achieving temperature control with functional group display of polymer nanoparticles. We used the carbohydrate d-mannose, which is known to bind with lectin protein concanavalin A (Con A), as a functional group. A mannose unit was conjugated to PNAM-*b*-PTAm micelles with a mannose-functionalized corona. Addition of the complementary polymer chain PNIPAM-*b*-PAAm to these micelles afforded mixed-corona micelles in which the mannose groups were buried inside the PNIPAM corona and showed no interaction with Con A at 25 °C, whereas above the LCST the PNIPAM chains were collapsed, revealing the mannose ligands bound with Con A proteins.

This base-pair-interaction-mediated “grafting to” approach was further explored with cellulose-grafted complementary-nucleobase-functionalized bottlebrush polymers to fabricate a series of polymer nanostructures with similar shapes but varying surface charge as well, to achieve thermally induced reversible morphological transformation from a sphere to a worm.^38^

## Nucleobase-Functionalized Polymers for Templated Synthesis

Templated synthesis is a fundamental biological process in nature, where the information stored in DNA strands in terms of sequence and spatial arrangement of the nucleic acids/nucleobases can be transferred precisely to its daughter strands. To mimic this phenomenon, nucleobase-containing polymers have been implemented to prepare precision polymers such as sequence-controlled polymers or polymers with similar degree of polymerization (DP) and dispersity as their parent polymers.^39−41^

Lo and Sleiman^39^ demonstrated the templated synthesis of nucleobase-functionalized polymers by alignment of the adenine monomers on a thymine-containing block copolymer template by virtue of A:T intermolecular H-bonding interactions followed by Sonogashira polymerization. The daughter polymer was found to copy the chain length of the parent polymer and possess narrow polydispersity, whereas similar polymers prepared by a non-templated method generated short oligomers with higher polydispersity. The success of this nucleobase-templated polymerization stems from excellent programmability due to the highly directional complementary nucleobase interaction.

**Figure 7:**
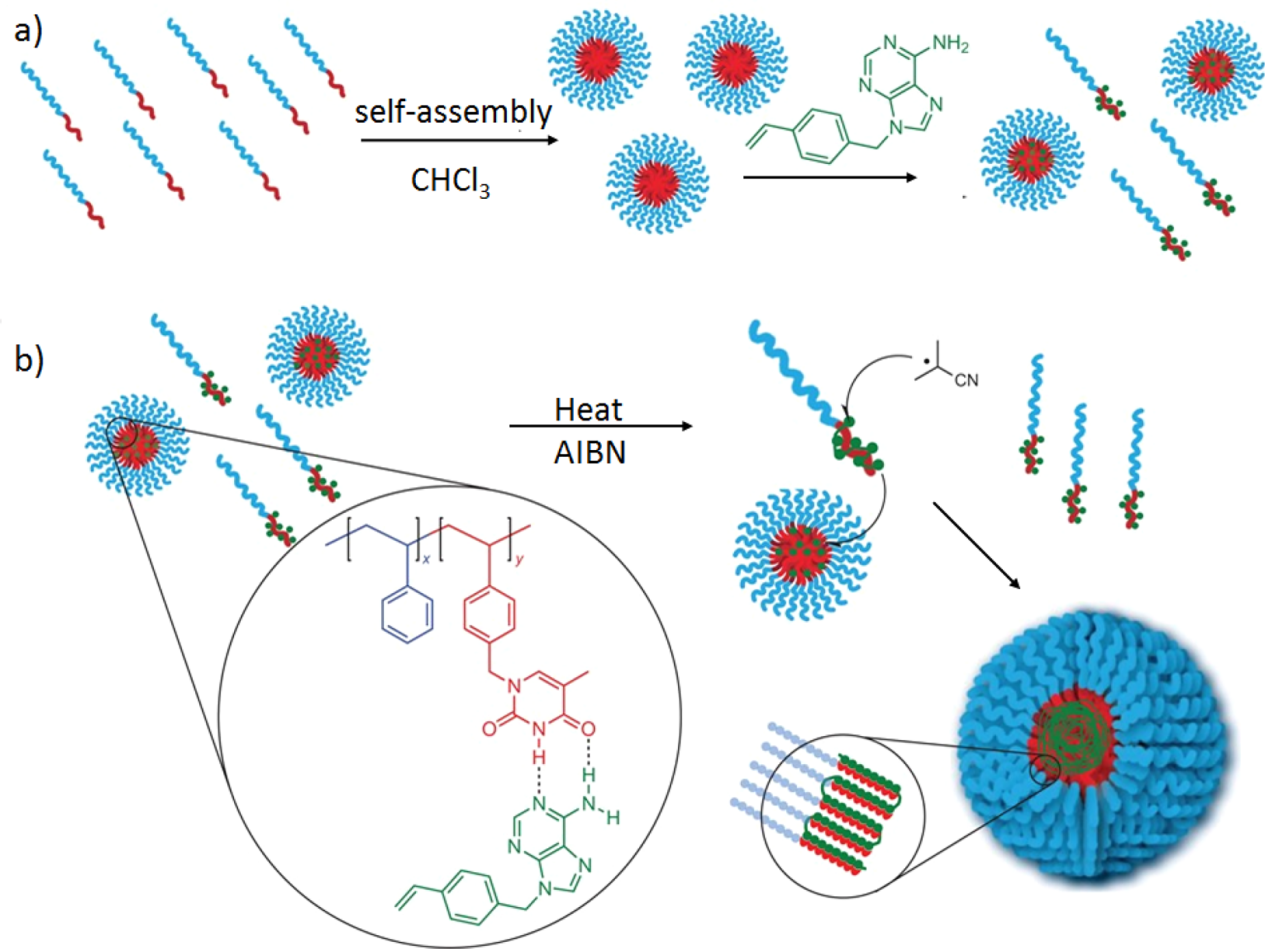
Schematic representations of (a) the self-assembly of the block copolymer PSt-*b*-PVBT into core–shell micelles and their dynamic exchange in the presence of a complementary monomer and (b) chain initiation on non-assembled polymer chains followed by propagation into the core of the micelles. Adapted with permission from ref (40). Copyright 2012 Nature Publishing Group.

We have combined self-assembly-mediated segregation of nucleobase units and a templating approach to synthesize polymers via free radical polymerization in order to obtain unprecedented control over the molecular weight of the daughter polymer (Figure 7).^40^ For this goal, the low-molecular-weight block copolymer poly(styrene-*b*-vinylbenzylthymine (PSt-*b*-PVBT) was synthesized to obtain core–shell micelles with thymine units at the core of the micelles that acted as a template to polymerize adenine-containing vinyl monomers. Herein, the micro confined zone in the micellar core enabled segregation of the propagating radical chains, leading to a complementary daughter polymer with high molecular weight (*M*_w_ up to ∼400 000 g mol^–1^) and low polydispersity (≤1.08). Afterward, such template-directed radical polymerizations were explored by Garcia et al.^41^ using uridine-derived polymer templates immobilized on a solid support to selectively achieve an adenine-containing daughter polymer within a mixture of different nucleobase-containing monomers.

## Toward the Conception of Nanomedicine: Stimuli-Responsive Delivery Vehicles

Formation of nucleobase-mediated cross-linked polymer networks has been proven to be a promising approach for drug delivery applications because it has several advantages, such as increased micellar stability, enhanced cellular uptake, increased drug loading capacity, reduced dye leakage, and stimuli-responsive controlled release properties.^42−48^ For example, Zhu and co-workers^42^ exploited adenine-terminated PCL (PCL-A) and uracil-terminated PEG (PEG-U) to fabricate amphiphilic supramolecular block copolymers that subsequently formed micelles. The drug release profile of the encapsulated doxorubicin (DOX) revealed enhanced DOX delivery at mildly acidic pH compared with that at physiological pH due to disruption of the H-bonding between the A:U pair. In a related study, Huang and co-workers^43^ reported dual pH-responsive supramolecular micelles using nucleobase-conjugated dextran polymers assembled via A:T interactions that disassembled at lower pH because of disruption of the complementary H-bonding as well as hydrolysis of the ketal group of the dextran backbone. Recently, Chen and co-workers^44^ reported the formation of bionic nanocapsules designed by cross-linking of thymine- and adenine-containing polymer precursors and demonstrated the role of nucleobase interactions in preventing premature drug leakage in their in vivo circulation in addition to the controlled diffusion of the encapsulated drug.

Nucleobase-functionalized non-linear polymers have also been explored as drug delivery vehicles.^45,46^ For instance, an adenine-terminated star-shaped PCL polymer and monofunctionalized uracil-connected PEG chain (U-PEG) were utilized by Zhu and co-workers to form a supramolecular amphiphilic hyperbranched copolymer that assembled into pH-responsive micelles.^45^ Subsequently, they further explored the assembly of A-functionalized brush copolymers with U-PEG that coassembled via A:U interactions to form polymer nanoparticles with pH- and salt-responsive properties in order to utilize this as a drug delivery vehicle.^46^

## Nucleobase-Interaction-Assisted Therapeutic Agent Loading

Another interesting strategy has been explored in the development of drug delivery systems by the use of a nucleobase-conjugated drug along with a complementary-nucleobase-conjugated polymer chain. For instance, Cheng and co-workers recently reported a coassembled system using uracil end-capped poly(propylene glycol) (U-PPG-U) and adenine-modified rhodamine 6G (A-R6G) featuring an extremely high A-R6G loading and excellent structural stability in biological media along with pH-triggered release of A-R6G.^49^ A similar concept was also explored by Lee and co-workers^50^ using uracil-functionalized PPG and an adenine-connected model drug. In that study, the formation of supramolecular micelles by U:A interactions followed by irradiation-mediated photo-cross-linking of the U units resulted in long-term structural stability of the micelles in serum solution.

**Figure 8:**
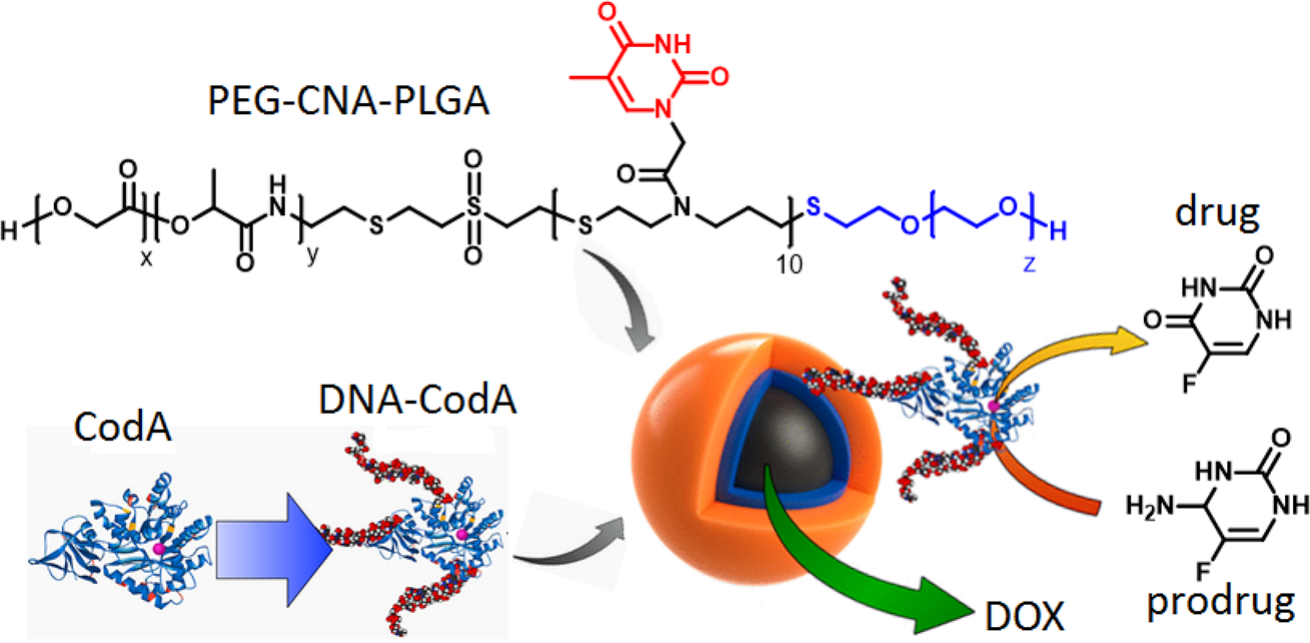
Structure of PEG-CNA-PLGA polymer and graphical representation of loading of a DNA-conjugated prodrug-activating enzyme. Adapted from ref (52). Copyright 2019 American Chemical Society.

Cha and co-workers synthesized a unique designed polymer (PEG-T10-PLGA) to use as a gene delivery vehicle.^51^ A central thymine oligomer (T10) connected to a hydrophobic poly(lactic-*co*-glycolic acid) (PLGA) block and a hydrophilic PEG block assembled into core–shell micelles where the middle T10 block was utilized to bind complementary DNA strands with high loading capacity. They further used this polymer to encapsulate a prodrug-activating enzyme, cytosine deaminase (CodA), connected with a complementary DNA strand by intermolecular H-bonding along with DOX via hydrophobic interactions and demonstrated the advantage of dual encapsulation on anticancer activity (Figure 8).^52^

## DNA-Inspired Self-Healing and Adhesive Materials

Self-healing materials are ubiquitous in nature and play a critical role in repairing damage and replacing degenerated parts in living organisms. For example, DNA repairs itself by radical scission of its strand, inducing a DNA repair cascade. This has been employed as a source of inspiration for the development of self-healing polymeric materials^53,54^ using the complementary and reversible intermolecular H-bonding ability of the nucleobase functionality.

In this context, Gu and co-workers^53^ developed a series of self-healing nucleobase-functionalized hyaluronic acid-based hydrogels derived from G:C intermolecular interactions. The G:C-interaction-mediated hydrogels showed better mechanical properties and higher healing efficiencies than the corresponding single-component (G or C) hydrogels, confirming the significance of the complementary H-bonding interaction. In a different design, Jiang and co-workers^54^ produced a type of self-healing polymer hydrogel with improved elasticity, tensile strength, and recoverable mechanical property by conjugating nucleobase (A and T) precursors to the known elastomer bis(3-aminopropyl)-terminated polydimethylsiloxane.

**Figure 9:**
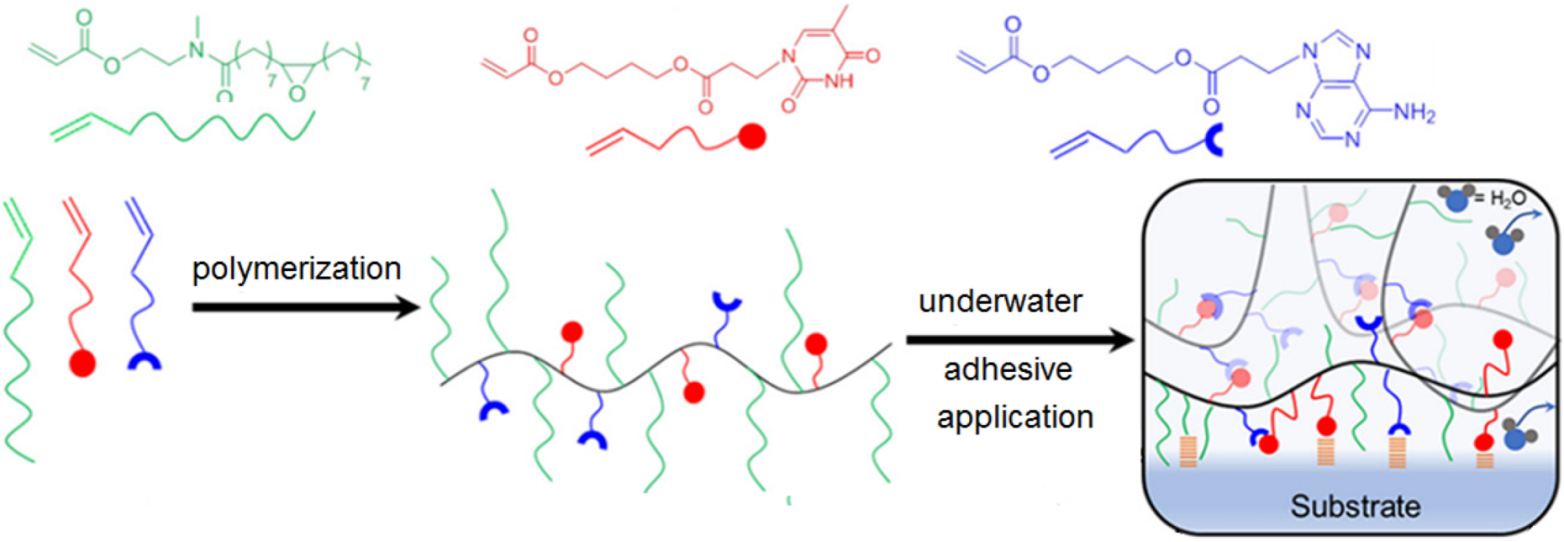
Schematic representation of the supramolecularly cross-linking hydrogel and the mechanism of adhesive performance induced by the nucleobase. Adapted from ref (58). Copyright 2022 American Chemical Society.

Adhesion is another common property of living organisms, such as mussels clinging on rocks and geckos walking on vertical walls. As various non-covalent interactions are responsible for such adhesive properties, researchers have been interested in exploring adhesive materials based on nucleobase-pair interactions.^55−58^ Long and co-workers^55^ prepared nucleobase-containing statistical copolymers by polymerizing A/T-functionalized acrylate monomers. Blending of A- and T-containing polymers led to supramolecularly cross-linked materials with tunable adhesive and cohesive strength depending on the A:T interaction. Yu and co-workers^56^ developed a new class of adhesive hydrogels prepared by introducing nucleobase functionality (A, T, and U) into the biodegradable polyphosphoester backbone. This nucleobase-tackified polyphosphoester adhesive gel showed excellent adhesive performance, controllable biodegradation, and outstanding biocompatibility due to multiple H-bonding interactions. Liu and co-workers^57^ developed a series of nucleobase (A and T)-functionalized homopolymers and statistical copolymers and demonstrated that compared with the individual homopolymers, a comixture of the complementary homopolymers exerted superior outstanding mechanical properties and higher adhesive propensity due to intra- and intermolecular H-bonding interactions. We have fabricated hydrophobic nucleobase-containing adhesives by synthesizing a random copolymer using hydrophobic long aliphatic chain-containing monomers as well as both A- and T-containing monomers (Figure 9).^58^ This elegantly designed supramolecularly assembled polymer network produced the strongest underwater adhesives reported to date, outperforming most previously reported underwater hydrogel-based adhesives.

## Conclusions and Perspective

As illustrated in this Account, nucleobase-functionalized molecular and macromolecular systems have proven to be a promising area of research bridging the gap between materials chemistry and biology. Base-pair interactions have been explored to drive self-assembly to fabricate a wide variety of structures ranging from spherical micelles^25,26,29,30^ to vesicles,^21,22^ one-dimensional fibers,^4,2,38^ helices, and even more complex three-dimensional^36,37^ structures, along with control of the functional design. These nucleobase-functionalized molecules and macromolecules have shown promising results in their prospective applications such as drug^42−48^ and gene^52^ delivery, templated polymerization,^39−41^ and superior adhesive^55−58^ and self-healing^53,54^ properties. The association constant of nucleobase interactions can be tuned from 10^2^ to 10^6^ M^–1^ as a function of several parameters such as the nature of the H-bonding unit,^37^ solvent polarity,^27^ temperature,^1,26^ pH,^45,46^ and number, sequence, and position of the nucleobases^31^ present in the system. Intermolecular interactions in these novel systems can be tuned upon requirement to create programmable assemblies featuring adaptive properties. Furthermore, with excellent performance in terms of responsiveness, tunable mechanical properties, biocompatibility, and biodegradability, these supramolecular materials certainly are expected to have many possibilities for the design and preparation of next-generation biomimetic materials.

Notwithstanding the aforementioned advantages, there are certain challenges that still need to be addressed in order to expand their impact and utility in biomaterials research. One such constraint is the often poor solubility of the nucleobases in many organic solvents, making it sometimes difficult to prepare new functional molecules or monomers. Second, resolving the poor solubility of nucleobase-containing molecules and macromolecules in aqueous solutions, which prevents scientists from exploring these nucleic acid analogues under physiological conditions, should be a next step forward to biomimicking. Exploration of new monomers connected to a suitable solubilizing group as well as synthesis of monomers with non-natural nucleobases or other complementary H-bonding systems such as diaminotriazine or ureidopyrimidinone can provide a feasible route to address this.

Built on the knowledge from the previous discussions, it is evident that investigations of nucleobase-containing substances have mainly been limited to A:T interactions and not much explored with G:C pairs. This could be due to undesirable interactions between G and C nucleobase monomers, which interact via a stronger three-point H-bonding, leading to monomer aggregation and poor polymerization control with large polydispersity and incomplete polymerization of the monomer. This issue could be overcome by protection of the nucleobases during polymerization followed by deprotection or by the use of postpolymerization functionalization techniques in order to fully explore supramolecular systems with G:C complementary pairs. Moreover, judicious incorporation of both A:T and G:C pairs in synthetic building blocks could open an exciting path for future research.

Looking forward, nucleobase-functionalized molecules and macromolecules are an intriguing platform to prepare complex nanostructures. However, it is worth emphasizing that we are still far from devising highly programmable multifunctional complex nanostructures like those present in nature. For instance, accurate positioning of α-helixes and β-sheets followed by macromolecular folding in a specific way to generate specific tertiary and quaternary structures in proteins is responsible for the transfer of molecular information necessary to play a large range of functions in our body. To harness such information transfer with wholly synthetic systems, one needs to judiciously design the constituent building blocks for logical reprogramming of hierarchical molecular organization. On that account, more research must be conducted to rationally derive engineered nanostructures by combining the molecular recognition properties of nucleobases with other directional interactions along with responsive functional groups in order to introduce multiple switchable properties. Additionally, more sophisticated macromolecular structures such as sequence-controlled polymers can be used, which can effectively control the chain folding or single-chain manipulation because of the high structural precision of the position of a specific nucleobase functionality. New approaches and strategies are required to produce sequence-controlled polymers, as to date genetically encoded sequence-defined polymers are limited only to polymerase- and ribosome-mediated synthesis, which imposes constraints on structural diversity. More importantly, these sequence-defined polymers need to be explored to construct more complex supramolecular nanostructures with high programmability and adaptable properties, which could prove to be a major breakthrough in supramolecular biomaterials research.
